# GATA: a graphic alignment tool for comparative sequence analysis

**DOI:** 10.1186/1471-2105-6-9

**Published:** 2005-01-17

**Authors:** David A Nix, Michael B Eisen

**Affiliations:** 1 Department of Molecular and Cell Biology, University of California, Berkeley, CA 94720, USA; 2 Department of Genome Science, Life Science Division, Lawrence Berkeley National Laboratory, Berkeley, CA 94720, USA

## Abstract

**Background:**

Several problems exist with current methods used to align DNA sequences for comparative sequence analysis. Most dynamic programming algorithms assume that conserved sequence elements are collinear. This assumption appears valid when comparing orthologous protein coding sequences. Functional constraints on proteins provide strong selective pressure against sequence inversions, and minimize sequence duplications and feature shuffling. For non-coding sequences this collinearity assumption is often invalid. For example, enhancers contain clusters of transcription factor binding sites that change in number, orientation, and spacing during evolution yet the enhancer retains its activity. Dot plot analysis is often used to estimate non-coding sequence relatedness. Yet dot plots do not actually align sequences and thus cannot account well for base insertions or deletions. Moreover, they lack an adequate statistical framework for comparing sequence relatedness and are limited to pairwise comparisons. Lastly, dot plots and dynamic programming text outputs fail to provide an intuitive means for visualizing DNA alignments.

**Results:**

To address some of these issues, we created a stand alone, platform independent, graphic alignment tool for comparative sequence analysis (GATA ). GATA uses the NCBI-BLASTN program and extensive post-processing to identify all small sub-alignments above a low cut-off score. These are graphed as two shaded boxes, one for each sequence, connected by a line using the coordinate system of their parent sequence. Shading and colour are used to indicate score and orientation. A variety of options exist for querying, modifying and retrieving conserved sequence elements. Extensive gene annotation can be added to both sequences using a standardized General Feature Format (GFF) file.

**Conclusions:**

GATA uses the NCBI-BLASTN program in conjunction with post-processing to exhaustively align two DNA sequences. It provides researchers with a fine-grained alignment and visualization tool aptly suited for non-coding, 0–200 kb, pairwise, sequence analysis. It functions independent of sequence feature ordering or orientation, and readily visualizes both large and small sequence inversions, duplications, and segment shuffling. Since the alignment is visual and does not contain gaps, gene annotation can be added to both sequences to create a thoroughly descriptive picture of DNA conservation that is well suited for comparative sequence analysis.

## Background

The most widely used methods for aligning DNA sequences rely on dynamic programming algorithms initially developed by Smith-Waterman and Needleman-Wunsch [1,2]. These algorithms create the mathematically best possible alignment of two sequences by inserting gaps in either sequence to maximize the score of base pair matches and minimize penalties for base pair mismatches and sequence gaps. Although these methods have proven invaluable in understanding sequence conservation and gene relatedness, they make several assumptions. One of their assumptions in generating the "best" alignment is that sequence features are collinear. For example, segments X, Y, Z in sequence one are also ordered as X, Y, and Z in sequence two. Another assumption is that short segments, like Y, have not become inverted or duplicated (e.g. X, Y, Y', Z). These rearrangement events are prone to be gapped out in dynamic programming and thus described as unrelated. Local alignment algorithms can be used to identify these rearrangements provided an exhaustive search is performed, but typically, only the highest scoring local alignments are considered valid and other, lower scoring local alignments are assumed to be spurious matches between unrelated sequences.

When aligning protein coding sequences, dynamic programming works quite well. Evolution exerts significant functional constraint on protein coding sequences. When an inversion, duplication or segment-shuffling event occurs, the protein is often compromised by truncation due to the introduction of frame shifts and stop codons. These deleterious mutations are typically lost and not observed in the surviving population. When aligning this type of constrained sequence element, dynamic programming works quite well.

Functional non-coding sequences do not appear to be as constrained in the ordering of elements as protein coding sequences [3-6]. Compact cis-regulatory modules, for example, enhance or suppress eukaryotic gene expression in response to external stimuli and play key roles in development and differentiation. One of the best characterized eukaryotic enhancers is the even-skipped stripe 2 element in Drosophila that controls transcription of the second transverse stripe of even-skipped mRNA during embryogenesis. Functional and comparative sequence analysis of stripe 2 clearly demonstrate that the enhancer maintains its specific activity across species yet displays significant small-scale insertions, deletions, and rearrangements of transcription factor binding sites within the module [7,8]. Tracing the evolutionary path of such non-coding elements is proving difficult with current alignment tools and may be assisted by a visual alignment program like GATA.

## Implementation

GATA employs a two tiered architecture in aligning DNA sequences. GATAligner executes and processed BLASTN output. GATAPlotter displays the processed alignments and annotation from GATAligner.

### GATAligner

**Figure 1:**
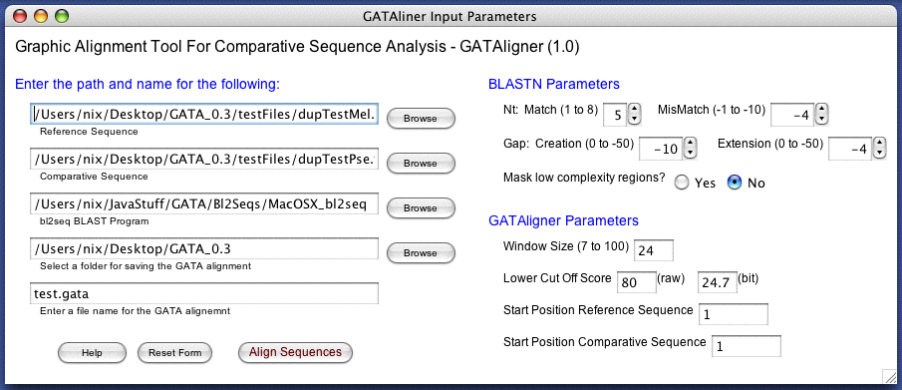
Screen capture of the GATAligner program.

The GATAligner application (figure 1) uses the NCBI bl2seq and BLASTN programs [9,10] to generate all possible local alignments between two input DNA sequences that score above a very low cut off (see Table 1). To avoid problems associated with visualizing both large and small local alignments, see Results/ Discussion, a sliding window is advanced at one base intervals across each local alignment. Windowed sequences scoring above a defined score are saved. To reduce the number of windowed sequences, each is compared to its neighbours and joined if they are of the same score and orientation. The score is not changed. These "sub-alignment" objects contain several features: a score, an orientation, a reference to the parental local alignment, the aligned sequences, and start and stop coordinates for each sequence. The sub-alignments are then saved to disk. This alignment and post-processing takes less than a minute for two 50 kb sequences using a window size of 24 and a score cut off of 25 bits on an 800 MHz PowerPC laptop computer.

Our initial goal was to create a high resolution sequence alignment and visualization tool to use in identifying small sequence rearrangements, like those associated with evolving non-coding regulatory DNA. We initially divided the first sequence into overlapping windows offset by one base pair. A Smith-Waterman dynamic programming algorithm was then used to align each window against the entire second sequence. Windows were scored, merged, and saved as above. Although this method is more rigorous than using BLASTN, it took 20–50 times as long, and did not produce significantly different results (data not shown). It should be noted that BLASTN requires seven consecutive identical bases to align two sequences. Thus in rare cases, some windows will be missed, for example, GGGGGGcTTTTTTaCCCCCCgAAAAAA versus GGGGGGaTTTTTTgCCCCCCtAAAAAA.

### GATAPlotter

**Figure 2:**
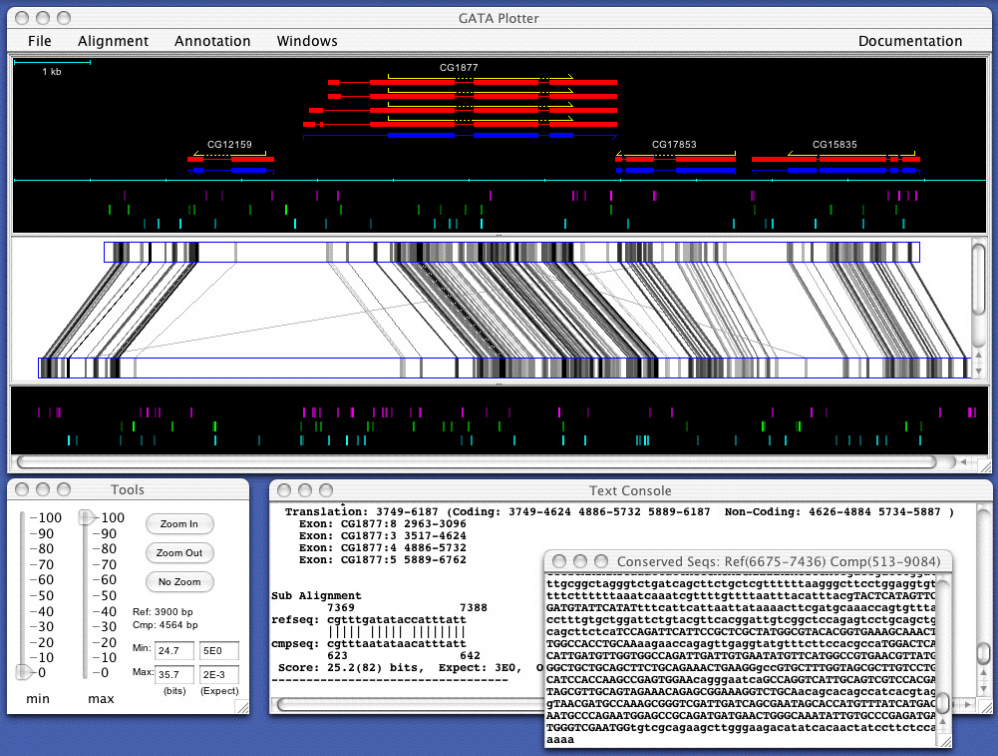
**Screen capture of the GATAPlotter program. **An alignment between *D. melanogaster *and *D. pseudobscura *surrounding gene CG1877.

The GATAPlotter application (figure 2) takes sub-alignment objects created by GATAligner and displays them graphically. Two boxes connected by a line are used to represent each sub-alignment. The boxes are plotted against horizontal representations of the input sequences with the reference sequence on top. The size of each box is determined by the start and stop positions in the sub-alignment. The shading of the boxes and connector line are scaled according to the sub-alignment score where solid black represents the highest score obtained, light grey the lowest. Lastly the colour of the connecting line is used to indicate the sub-alignment orientation, black for +/+, red for +/-. Where windows overlap, those with the highest score are displayed on top. Single clicking on overlapping windows retrieves all of the underlying windowed sequence alignment information. Double clicking fetches all of the associated local alignment information as parsed from BLAST.

**Figure 3:**
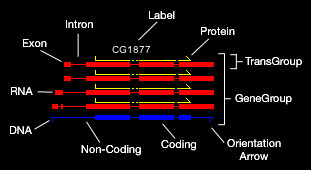
**Rendered gene annotation in GATA. **A typical protein coding gene is visualized as a GeneGroup comprised of multiple TransGroups containing Exons, Introns, and a Protein transcript. Arrows designate orientation. The DNA glyph is rendered as both Non-Coding and Coding elements.

GATAPlotter also has the capability to display extensive gene annotation for one or both input sequences. The principle component of gene annotation rendering by GATA is the "GeneGroup" (figure 3). Each GeneGroup is drawn independent of other GeneGroups and is allowed to float within the panel to avoid overlap. A typical GeneGroup contains one DNA sequence from which one or more "TransGroups" are derived. Each TransGroup contains exons, an RNA transcript and possibly a protein translation. Each of these features are described using the Berkeley Drosophila Genome Project GFF format [11]. Coding and non-coding DNA sub features are only created in the presence of translation features and represent the most conservative estimation of what is protein coding sequence. If any translation predicts a larger coding region than the others, this is adopted for the entire GeneGroup. A point of confusion by many is that exons encode protein peptides. This is not necessarily true (i.e. 5' and 3' UTRs) and has lead to a variety of annotation rendering errors. When parsing a GFF file, GATAPlotter looks for the following GFF features: exon, translation, transcript, *gene*, *rna*, *transpos*, *misc*, where an * represents one or more wild cards. These wild card "genes" are interpreted as the closing feature on the GeneGroup from which all the proceeding TransGroups are derived. Annotation for both strands is drawn together; arrows are used to indicate orientation. Features not recognized by the parser are interpreted as novel user defined elements and rendered in their own tracks. GFF annotation examples, templates, and extensive descriptions are provided under the GATAPlotter "Documentation" menu. See table 2 and 3 for a complete listing of program options.

## Results and discussion

**Figure 4:**
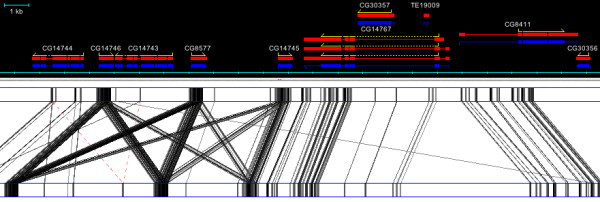
**Example: gene triplication. **An example of a gene triplication in *D. melanogaster *and *D. pseudobscura *surrounding gene CG14745

**Figure 5:**
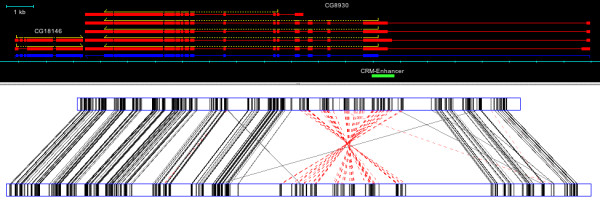
**Example: sequence inversion. **An example of a sequence inversion event between *D. melanogaster *and *D. pseudobscura *surrounding gene CG8930.

To illustrate the types of rearrangements GATA can distinguish, examine figures 4 and 5. Both contain alignments between *Drosophila melanogaster *and *D. pseudoobscura*. Figure 4 contains three highly similar genes. Figure 5, inversions of putative enhancers. Annotation for each was obtained from whole_genome_annotation_dmel_RELEASE3-1.gff [11]. Orthologous sequences were isolated using the FlyCatcher program [12]. In cases where alignment windows overlap, the lowest scoring windows are drawn first and higher scoring windows placed on top. Both, connecting lines and their associated boxes are shaded according to score.

Several related alignment and visualization tools have proven useful in comparative sequence analysis. Dot plot analysis can be used to identify duplications and inversions. Programs such as Dotter, JDotter, Dotlet, and Family Relations [13-16] generate graphical representations of sequence conservation by scoring identity between two perpendicular sequence representations. Although, mapping annotation to dot plots containing duplications and inversions is rather difficult and counter intuitive. Programs such as Artemis/ACT, LALNVIEW and to some extent, PLALIGN [17-20], utilize alignment information generated from dynamic programming algorithms to create box-line-box representations of each local alignment. These are similar to GATA but do not divide local alignments into window scored sub-alignments. This is unfortunate since window scoring enables a more detailed view of the actual sequence similarity within a large local alignment. Moreover, meaningful visualization using these browsers requires setting a high cut off score for the visualized local alignments. This effectively eliminates smaller, lower scoring local alignments that may provide alternative or even better inverted local alignments. GATA's windowed post-processing overcomes these associated problems.

One program that is proving quite useful in avoiding the collinearity problem while still using a dynamic programming algorithm is Shuffle LAGAN [21]. Alignments generated by Shuffle LAGAN are combine with alignment annotation viewers such as VISTA [22,23] to align entire genomes. K-BROWSER/ MAVID and Mauve are two additional genome browser/ aligners that look equally promising [24-26]. Although, it should be noted, these programs are designed to provide genome wide alignments and identify large-scale rearrangements, GATA is best suited at interrogating non-coding DNA sequences between 0–200 kb in size for both large and small rearrangements.

One of the major challenges facing bioinformaticians is the development of alignment and visualization tools for multi-species comparative sequence analysis. Within the fly community alone, 12 divergent species of diptera and hymenoptera will be sequenced within 3 years. A variety of higher eukaryotes including human, mouse, rat, dog, chimp, cow, chicken, opossum, and platypus have or are in the process of being completely sequenced. How can one visualize the alignment and species-specific annotation for 12 orthologs of a particular gene or a genomic segment? The GATA alignment paradigm is well suited to this challenge and will play a prominent role in GATA's development.

## Conclusions

As comparative sequence analysis accelerates, scientists need more sophisticated alignment and visualization tools to define the evolutionary relationships and functional significance between particular orthologous sequences. This is especially true for regulatory, non-coding DNA that can show significant small-scale rearrangements. These new tools must incorporate detailed annotation alongside views of sequence conservation while providing easy access to the underlying sequence information. GATA provides one such solution.

## Availability and requirements

Project name:

GATA, graphic alignment tool for comparative sequence analysis.

Project home pages:

 and 

Operating system(s):

Platform independent

Programming language:

Java

Other requirements:

Java 1.4 or higher

License:

GNU GPL

Any restrictions to use by non-academics:

None

## Authors' contributions

DAN designed and constructed the GATA programs with advice and supervision from MBE.

## Figures and Tables

**Table 1:** GATAligner parameters and features

**NCBI-BLASTN Parameters**	
*Nucleotide Match*	Score added to the total for each match.
*Nucleotide Mismatch*	Score subtracted from total for each mismatch.
*Gap Creation*	Score subtracted from total for each new gap.
*Gap Extension*	Score subtracted from total for each additional base in a gap after its creation.
*Low Complexity Mask*	Use of DUST to mask and thus not align regions of low complexity.
	
**GATAligner Parameters**	
*Window Size*	Size of window used to score sub-alignments in each local alignment. Sub-alignments smaller than the window size will be saved provided they are at or above the score cut off. When aligning longer sequences, increase the size of the window as well as the cut off score to minimize non-related alignments.
*Score Cut Off*	Score (raw or bits) at which windowed sub-alignments are saved or discarded. The higher this score is set the faster GATAligner will run. Set between 20–25 bits for a window size of 24 when aligning sequences less than 10 KB. For larger sequences, increase the cut off and window size (e.g. 30 bits and 30 bp).
*Start Positions for Reference and Comparative Sequences*	Use this to maintain register with gene annotation.
*Multithreaded*	GATAligner is multithreaded. Queue up multiple alignments.

**Table 2:** GATAPlotter menus

**File-Menu**	
*Open or Close Alignments*	Open a new GATA plot or close the present GATA plot.
*Quit*	Quit the entire application.
*Save GATAPlot Image*	Use to save a high resolution PNG file of the GATAPlot.
*Save GATAPlotter Settings*	Select this menu option to save the current settings. These will be used upon opening new GATA plots. Generic Track settings are not saved. To restore the defaults, select the Redraw Using Defaults from the Windows menu and then the Save GATAPlotter settings. Alternatively, delete the GATAPlotterPreferences file in the GATA folder.
	
**Alignment-Menu**	
*Sizing parameters*	A variety of parameters to change the height, width, thickness and relative location of the Alignment panel shapes.
*Set Nucleotides Per Pixel*	Allows for specifying the number of nucleotides that are rendered per pixel enabling size synchronization between different GATAPlots.
*Fetch Conserved Sequences*	Use this option to reformat both the reference and conserved sequence using the visible alignment boxes in the GATA plot. Upon selection, a dialog box will appear asking how you would like to reformat the non-boxed sequences. These non-conserved sequences can be replaced with any single character (e.g. N or X) or converted to lower or upper case. Use the sliders in the Tools Panel to adjust what is visible.
*GATAligner Parameters*	Select to retrieve all the GATAligner settings used in making the GATA alignments and GATA plot. (e.g. score cut off, window size, match, mismatch, etc).
	
**Annotation-Menu**	(These menu items are only available if gene annotation has been added to the GATA plot.)
*Gene Groups*	Use to hide or show all of the gene groups (Protein, RNA, DNA) or labels.
*Protein, RNA, DNA*	Select whether to hide, show or change their colour.
*Scale Ruler*	Select to hide or show, change the colour, or move the scale bar.
*Tracks*	This option contains global effectors for generic tracks.
*RefTrks/ CompTrks*	If generic features are found within the GFF file, each is assigned its own track. Their thickness, colour, visibility, and label visibility can be modified using the appropriate options.
*Pix Btw*	A variety of adjustments can be made to the number of pixels that are placed between features. Negative numbers are valid if you want to overlap features.
*Line Thickness*	Line thickness can be set to control the size of Protein, RNA, and DNA features.
*Colour*	The background panel and label colour can be set using these options.
*Scale Track Colours By Score*	If generic tracks have been generated and are associated with a score, they can be shaded using the scaling feature. Select the method GATA should us to convert the reported scores to linear numbers. (e.g. Often hits to a position weight matrix are scored in log units. Select the appropriate base log 10, log 2, or natural log.) After converting the scores for a particular track, a range is estimated and used to adjust the opacity of each feature from 30% for the lowest scoring feature to 100% for the highest scoring feature. This allows visual comparison of features within a track. Comparisons between tracks are only valid if they have the same range.
	
**Windows-Menu**	
*Show All*	Retrieves and displays all hidden windows.
*Hide All*	Hides all windows except the main GATAPlotter alignment window.
*ReDraw Using Defaults*	Redraws all panels using GATAPlotter default values.
	
**Documentation-Menu**	Extensive documentation for GATA including examples.

**Table 3:** GATAPlotter windows and features

**Tools Window**	
*Score Sliders*	Sliders can be used to control the minimum score and maximum score used in deciding which sub-alignment box-line-boxes are displayed. The units are a normalized range where zero is set to the value assigned in GATAligner to the Lower Score Cut Off. 100 is set to the value obtained by multiplying the Window Size by the Nucleotide Match. The actual window bit and Expect values set by the sliders are shown in the adjacent boxes. Since the shading is relative to these minimum and maximum values, be careful in making comparisons in shading between two GATA plots. Such comparisons are only valid if the same window size, cut off score, and scoring scheme were used. Check the actual score by clicking on the shaded box or connecting line to see the real bit score. (Bit scores are scoring system independent and can be used to directly compare alignments. Raw scores are relative to the settings for match, mismatch, gap, etc.)
*Zoom Buttons*	The zoom buttons allow for zooming in and out.
*Ref or Cmp*	These numbers report the position of the mouse, in base pairs, when the mouse passes over one of the sequence bars.
	
**Mouse Clicks**	Single clicking a gene annotation feature retrieves and displays all information associated with that feature in the Text Console Window. Likewise single clicking an alignment box or line displays the sub-alignment information. Double clicking fetches the sub-alignment and its parental local alignment. The sub-alignment is indicated by the asterisks in the larger local alignment. All visible alignments beneath a mouse click are retrieved. Use the Score Sliders to determine which boxes are visible.
	If you are interested in a sub section of the alignment, drag the mouse over the region and a reformat box will appear. If you drag the mouse over one sequence and it contains box-line-boxes, these will to used to fetch the corresponding sequence from the other sequence. If you drag the mouse over both sequences, sub sequence sections will be retrieve regardless of the location of box-line-boxes.
	
**Text Console**	A resizable scrolling container for text messages generated by mouse clicking.
